# Hepatocellular adenomas with severe intra-abdominal bleeding, related to an underlying coagulation disorder: a case report

**DOI:** 10.1186/s13256-024-04709-7

**Published:** 2024-08-13

**Authors:** C. A. J. Oudmaijer, J. Sprakel, D. Sprengers, K. A. Wiese, N. Vogelaar - Tintel, J. I. Franken, T. Terkivatan, R. J. Porte, J. N. M. IJzermans

**Affiliations:** 1 Erasmus MC Transplant Institute, Department of Surgery, Division of Hepatobiliary and Transplantation Surgery, University Medical Center Rotterdam, Office RG-220, Dr. Molewaterplein 40, 3015 GD Rotterdam, the Netherlands; 2 Princess Máxima Center for Pediatric Oncology, Utrecht, the Netherlands; 3 Oncode Institute, Utrecht, the Netherlands; 4 Department of Gastro-Enterology, Division of Hepatology, University Medical Center Rotterdam, Rotterdam, the Netherlands; 5 Department of Radiology, University Medical Center Rotterdam, Rotterdam, the Netherlands

**Keywords:** Hepatocellular adenoma, Treatment strategy, Intra-abdominal bleed, Case report

## Abstract

**Background:**

Hepatocellular adenoma is a rare benign liver tumor. Typically, hepatocellular adenomas are solitary and are found in young women who use estrogen-containing contraceptives. The occurrence of multiple hepatocellular adenoma has been linked to higher body mass index, and as the prevalence of overweight increases, multiple hepatocellular adenomas are seen more often. An hepatocellular adenoma does not always necessitate treatment, as they can regress under conservative strategies. In incidental cases, an adenoma presents owing to bleeding, which is mostly self-limiting. If it is not, embolization of hepatic involved vessels is indicated.

**Case presentation:**

In this case report, we discuss a 42-year old Caucasian woman with multiple hepatocellular bleeds, treated by multiple endovascular procedures. After the first embolization of an adenoma in the right liver lobe, a second bleed occurred in the left lobe, necessitating additional endovascular intervention. During admittance, treatment was complicated by pulmonary embolism and a pneumonia. During follow-up, our patient was diagnosed with antiphospholipid syndrome.

**Conclusion:**

Hepatocellular adenoma is a rare diagnosis that requires centralized expertise. This particular case illustrates the complexity of treatment strategies for associated intra-abdominal bleeding and possible complications. Although liver adenoma is often an incidental finding, it can also result in significant morbidity. Centralization of treatment leads to expertise in managing complex treatment strategies.

## Key Points

- Hepatocellular adenoma is a rare diagnosis with a complex treatment strategy. This strategy depends on symptoms, initial presentation, tumor size, molecular diagnostics, and patient-specific factors.
- A bleed from a liver adenoma requires a multidisciplinary approach and consultation with, or preferably referral to, an expertise center. Expertise is essential for making the appropriate diagnosis and initiating treatment.
- Concomitant diagnoses or complications can complicate the treatment of hepatocellular adenomas and require a multidisciplinary approach.

## Background

Hepatocellular adenoma (HCA) is a relatively rare benign solid tumor, often an incidental finding encountered during diagnostics for abdominal pain, and is typically found in women with prolonged use of oral contraceptives and/or obesity [1, 2]. The incidence is estimated at 30–40 diagnoses per million active contraceptive users [1], with a prevalence in the general population ranging between 0.001% and 0.004% [3]. As it is usually an incidental finding, unrelated to the symptoms the patient presents with, immediate medical intervention is not indicated [4]. Depending on adenoma size, characteristics on magnetic resonance (MRI), and molecular diagnostics, a surveillance period is often recommended [4–7]. During this period, oral contraceptives are discontinued, and weight reduction is advised [6, 8, 9]. This policy consists of a 6-month surveillance period after which regression is assessed by MRI [4, 6, 10, 11]. After this initial observation period, management will be based on patient characteristics, current tumor size, and the predicted chance of regression [4].

HCA consist of multiple subtypes, including inflammatory, steatotic, and β-catenin-mutation-associated lesions. Among these, the inflammatory and steatotic subtypes are most commonly observed in females [7, 12, 13]. Patients with inflammatory HCA usually have a higher body mass index (BMI) compared with patients with steatotic HCA [7, 12]. Additionally, inflammatory adenomas present more often as multiple lesions, whereas steatotic HCA are more likely to be single lesions [7, 12, 14]. If there is any doubt about the nature of the lesion, if it is very large or exophytic, or if a β-catenin mutation is found during molecular analysis, surgical intervention is recommended [4]. Bleeding from an HCA occurs almost exclusively in adenomas larger than 5 cm, and most often occur during pregnancy owing to growth during prolonged altered hormonal exposure [15]. Treatment for an acutely bleeding adenoma depends on bleeding characteristics and symptoms, but transarterial embolization has emerged as the treatment of choice [15–17]. In this case report, we discuss a patient with multiple bleeds, treated by multiple endovascular procedures.

## Case presentation

**Fig. 1:**
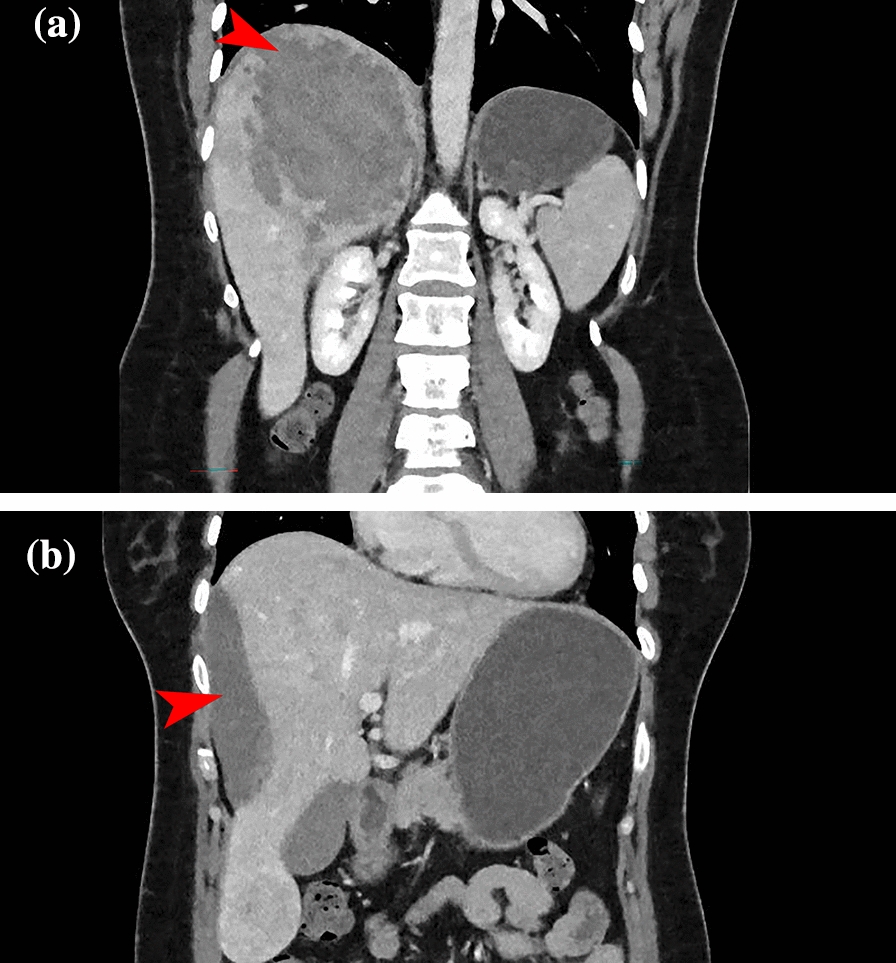
Initial abdominal computed tomography at presentation in the peripheral center, showing a large adenoma with post-bleeding status in segment 7 (**A**, marked with red arrow) and an extensive sub-capsular hematoma (**B**, marked with red arrow). No contrast extravasation is visible, indicating no active bleeding at the time of the computed tomography scan

Our patient, a 42-year old Caucasian woman, initially presented at a local hospital, with acute-onset abdominal pain. Her medical history included hay fever, irritable bowel syndrome, dysmenorrhea, and a depressive episode in the past. Active medication use consisted of cetirizine and ethinyl/levonorgestrel 30/150; she had been using the latter for over 20 years, and had been using it continuously for the past 10 years owing to dysmenorrhea. At the time of initial presentation, she also mentioned pain between the shoulder blades. Clinical examination showed diffuse tenderness in the abdomen, most prevalent in the right flank and right upper quadrant, and aside from a tachycardia of 115 beats/minute, further clinical examination was normal. Initial diagnostics focused on ruling out a pulmonary embolism, for which a chest computed tomography (CT) was performed. A pulmonary embolism was successfully excluded, but the radiologist noted a diffuse heterogeneous aspect of the right liver lobe. An additional abdominal CT revealed a subcapsular hematoma of 12 cm (Figure. 1), suspicious for a bleeding liver lesion. Furthermore, multiple hypervascular lesions scattered throughout the liver were found, most prominently in segments 2 and 5/6, consistent with the appearance of multiple adenomas. Serology showed no signs of active viral disease. Initially, a conservative approach was adopted given the confirmed intraparenchymal bleeding on CT, with no evidence of active bleeding. The patient was admitted for observation at her local hospital. During hospitalization, her pain worsened, and her hemoglobin levels dropped from 6.7 to 4.8 mmol/L. Consequently, consultation was sought with our academic center for transfer.

**Fig. 2:**
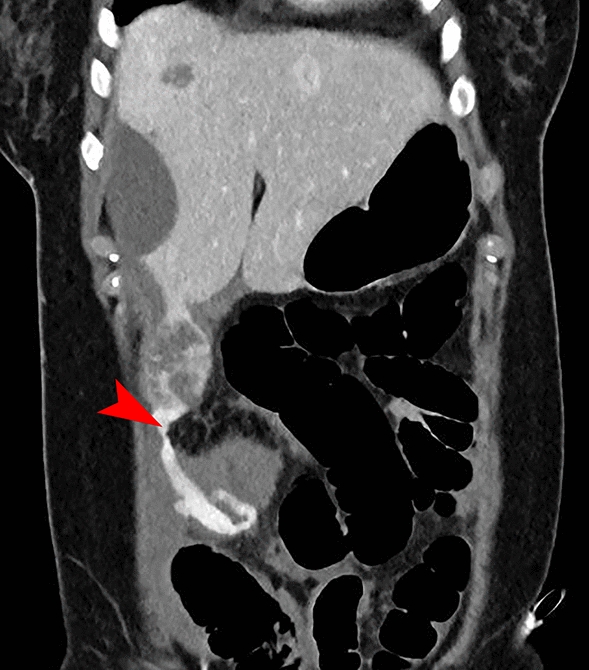
This abdominal computed tomography revealed a new active bleed from the adenoma in segment 6, with a newly developed extravasation (marked with red arrow) into the free abdominal cavity: the right paracolic region behind the right rectus abdominis muscle

**Fig. 3:**
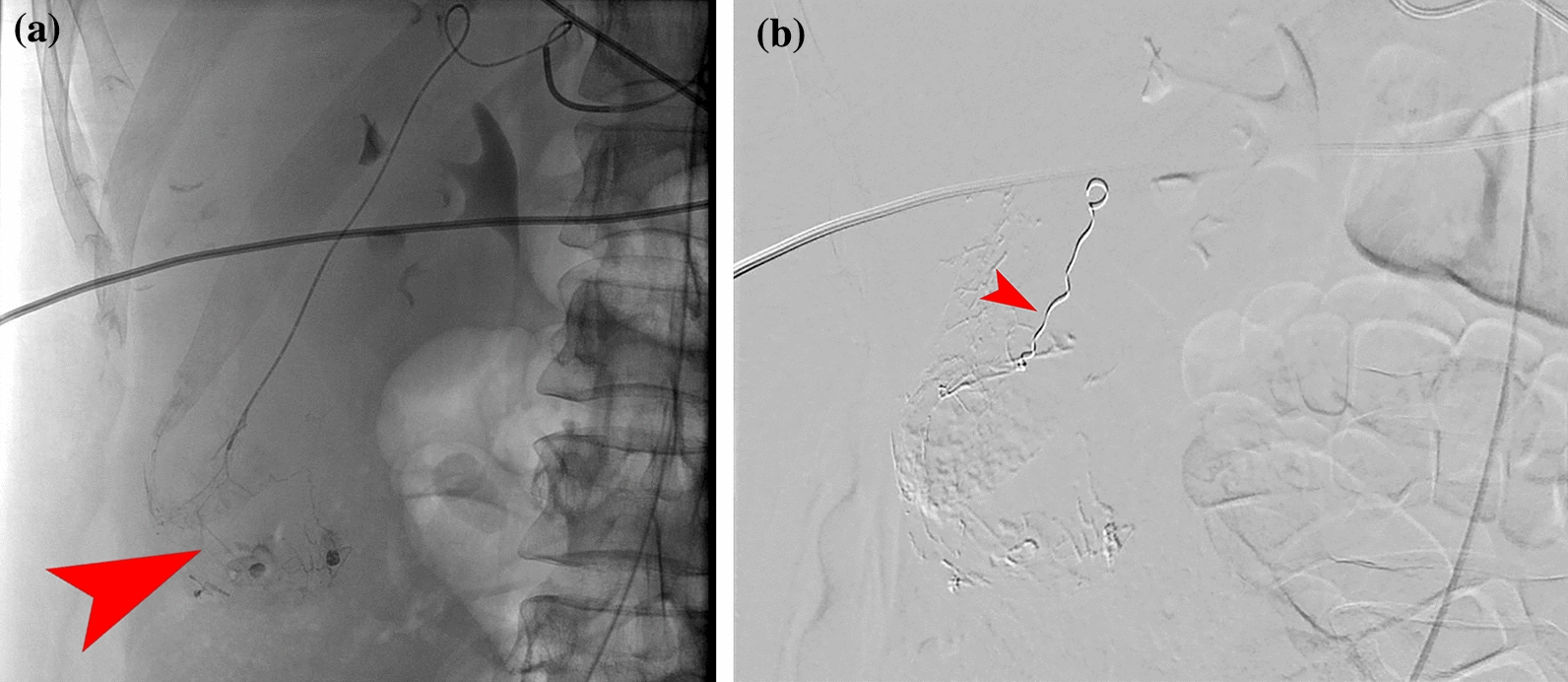
Images from the second angiography. **A** Angiography with selective injection of contrast into the arterial branch toward the adenoma in segment 5/6. Contrast extravasation is observed at the site of bleeding focus (marked with red arrow). **B** After treatment by embolization of two branches with histoacryl, followed by placement of a coil in the arterial feeder toward the adenoma for added assurance (marked with red arrow)

Upon arrival at our academic center, clinical examination was in accordance with what was described earlier. An additional 3-phase abdominal CT was performed, revealing an active source of the bleed at a lesion in segment 7 of the liver. Subsequently, selective embolization of two feeder arteries of the lesion in segment 7 was performed by our interventional radiologists using particles. The second lesion in segment 5/6 showed no signs of active bleeding and was therefore not embolized. On the following day, our patient developed hypovolemic shock, presenting with hypotension, tachycardia, and a further decline in hemoglobin. A bedside ultrasound showed free fluid in the abdomen, and owing to the suspicion of recurrent bleeding, a new 3-phase abdominal CT was performed (Fig. 2). This scan revealed a new active bleed from the lesion in liver segment 5/6, extending into the intra-abdominal space. Selective angiography confirmed an active bleed from the lesion in segment 5/6, for which selective embolization of two arterial feeders was performed using glue (histoacryl) and a coil (Fig. 3). Following the procedure, bleeding persisted from the catheterization site at the right femoral artery, which was compressed successfully.

**Fig. 4:**
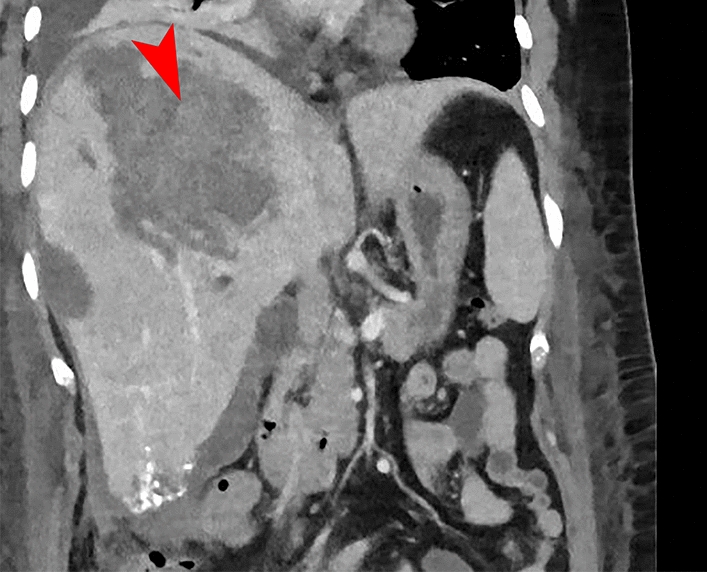
Abdominal computed tomography showing a recurrent/new bleed from the adenoma in segment 7 (marked with red arrow). Additionally, you can see the result of embolization with histoacryl of the adenoma in segment 5/6

At 2 days after the last radiological intervention, following stabilization of vital signs and hemoglobin, and the initiation of clinical recovery, the patient was transferred back to the referring hospital. However, after 2 days, a new decrease in hemoglobin was observed, and a repeat abdominal CT raised suspicion of recurrent bleeding from the lesion in segment 7 (Fig. 4). The patient was once again transferred back to our center for transarterial embolization by the interventional radiologist; angiography did not reveal a bleeding source, but given the recurrence of bleeding, a pragmatic decision was made to embolize the remaining tortuous feeders to the residual lesion using particles, followed by a thrombin injection at the pseudo aneurysm located in the right groin. A day after this intervention, the patient developed progressive dyspnea, and imaging studies confirmed the suspicion of bilateral segmental and subsegmental pulmonary embolisms, without signs of cardiac overload. Given the combination of recurrent bleeding and now a thromboembolic complication, the hematologist was consulted, who recommended therapeutic low molecular weight heparin (LMWH) treatment for prevention of further embolisms. Owing to the multiple bleedings, after thorough consideration, we decided upon a pragmatic intermediate dosage (twice daily 5700 IU).

**Fig. 5:**
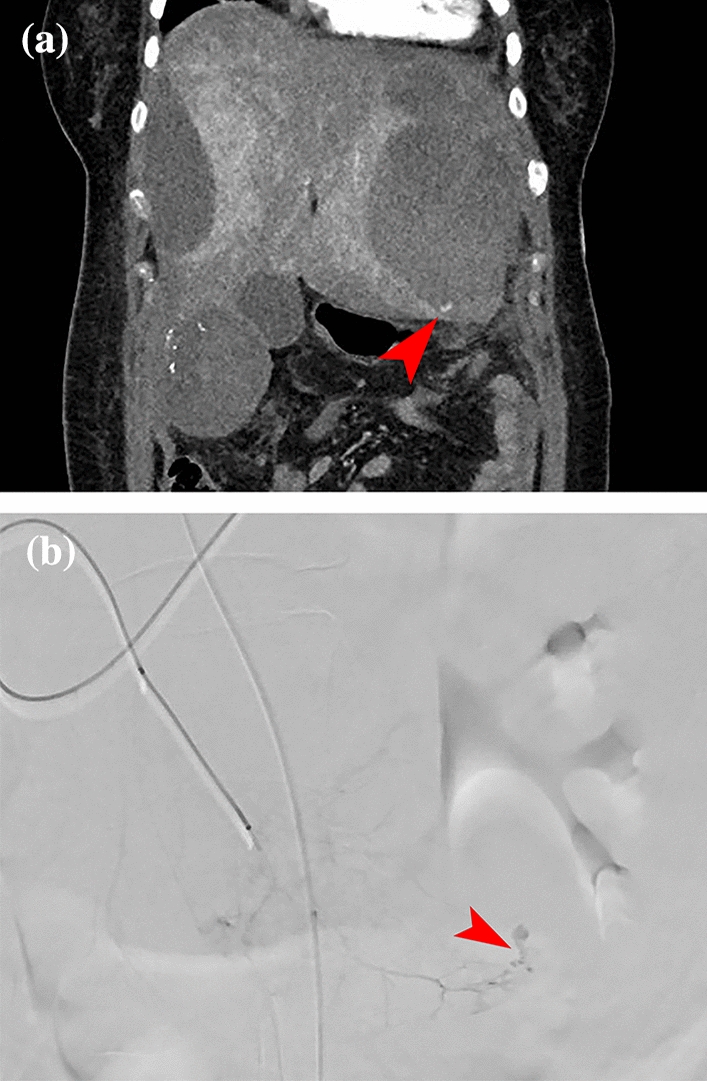
Images following persistent suspicion of recurrent bleeding. **A** Additional abdominal CT shows a small arterial bleed with concomitant hematoma, breaking through the capsule, resulting in a hematoma in the left paracolic gutter extending into the small pelvis (marked with red arrow). **B** The bleed as shown during selective catheterization (marked with red arrow)

After the initial two doses (evening and morning), a suspicion of recurrent bleeding or an infected hematoma arose owing to a significant increase in C-reactive protein, leukocytosis, and a recurrent decrease in hemoglobin. A new 3-phase abdominal CT showed encapsulation of the free fluid around the liver tip, but the primary finding was a new large subcapsular hematoma in the left liver lobe. Additionally, the CT showed an incipient pneumonia in the basal lung fields, with adjacent atelectasis and pleural effusion, both reactive to the previous diagnoses. Owing to the persistent suspicion of recurrent bleeding, we decided upon a 3-phase abdominal CT according to bleeding protocol and re-intervention (Fig. 5). Angiography revealed a small arterial blush in the subcapsular hematoma in segment 2/3 with breakthrough of the liver capsule along the paracolic gutter into the small pelvis, for which embolization of the blush was performed. Additionally, intravenous antibiotic treatment for pneumonia was initiated. Subsequently, placement of a vena cava filter was debated to prevent further embolisms migrating to the lungs, given the suboptimal anticoagulation therapy. After discussion, the vena cava filter was not placed owing to absence of evidence for deep vein thrombosis in the lower vessels or vena cava, and the dosage of LMWH was adjusted (twice daily 2850 IU), after which the situation stabilized. A week after the last intervention, 14 days after first admission (9 days after second admission) at the academic hospital, a reevaluation was performed: abdominal and chest CT showed a decrease in the subcapsular hematomas, reduction of the pulmonary embolisms, a slight residual pneumonia, and reduction of the reactive pleural effusion. Further recovery proceeded uneventfully, with intensive in-hospital counseling for mobility and physical rehabilitation. Ultimately, the patient was discharged to her home environment 30 days after her initial presentation, 21 days after the start of the second admission.

During follow-up, the patient’s clinical condition improved further. At 2 months after hospital discharge, she presented at the outpatient hematology clinic, where additional laboratory tests were performed, revealing abnormal coagulation results. At both time points, anticardiolipin immunoglobulin G (IgG) and IgM antibodies were increased, thereby conforming the prior suspicion of antiphospholipid syndrome (APS), for which further workup and treatment were initiated. The recommendation to adjust the current (high prophylactic) dose of LMWH to therapeutic for 3 months, was not followed owing to the patient’s fear of recurrent bleeding. A MRI of the liver confirmed the diagnosis of hepatocellular adenoma and showed regression of hematomas, persistent vital residual tissue of the embolized adenomas, and a few unchanged adenomas in segments 3 and 6. A surveillance period was agreed upon, with a MRI scheduled after 6 months, which showed further regression of the hematomas. Subsequently, LMWH was switched to a direct oral anticoagulant (rivaroxaban at 10 mg daily) after multidisciplinary discussion and careful consideration with the patient. This switch in medication was tolerated without any events.

## Discussion

The current case underscores the importance of vigilance to prevent the occurrence of serious complications during admittance for a bleeding hepatocellular adenoma. Concomitant comorbidities, such as severe coagulation disorders or hepatic steatosis with fragile liver parenchyma, should be considered in the decision-making process during the initiation of treatment, e.g., during trans-arterial embolization.

The rarity of the diagnosis underscores the importance of expertise in diagnostics, therapy, and follow-up. For accurate diagnosis and treatment of complex liver tumors, including adenomas as described in this case, close collaboration with specialized centers is essential. Transfer to such centers should be considered to access not only interventional radiology facilities but also expertise in liver surgery and intensive care [15–17]. The current case confirms the need for this expertise, given the poorly controllable bleeding episodes requiring several endovascular treatments, with our initial decision for transfer proving overly optimistic in retrospect. However, after initial diagnosis and successful initiation of therapy, follow-up can be conducted in the local setting in collaboration with the patient and the local hospital.

The current case also emphasizes the diagnostic dilemma during the acute presentation of the adenoma. Both the nature of the tumor and its actual size were unknown; given the active bleeding, the primary lesion could not be characterized. Contrast-enhanced MRI (e.g., with Primovist [18]) has no added benefit in the acute setting, where identifying and controlling the bleeding source is paramount. After hematoma resorption and decompression of the liver parenchyma, a MRI can provide further information about the type and number of lesions. Sometimes, an additional tumor biopsy is indicated to confirm the suspected diagnosis and perform molecular analysis [4].

Apart from the diagnostic dilemma, there is also a therapeutic dilemma during the acute phase. In the setting of parenchymal bleeding, if the patient is hemodynamically stable and there is no downward trend in hemoglobin, a conservative strategy is justified. If the bleed spreads into the peritoneal cavity or is a parenchymal bleeding with hemodynamic consequences, selective endovascular treatment is indicated [15–17]. Experience with similar cases is required, especially given the balance between the degree of embolization and nontarget embolization, with a greater risk of damage to healthy liver parenchyma [19]. Our case also illustrates that pulmonary complications are not uncommon in a bedridden patient with abdominal issues. However, the occurrence of a thromboembolic complication in a patient with a bleeding adenoma is unusual. It further complicated the therapeutic dilemma, given the recurrent bleeding and a recurrence of bleeding after the initial LMWH administrations. Initial presentation of APS via abdominal symptoms is very rare, as it often initially presents as (recurrent) deep vein thrombosis, thrombocytopenia, or complications during pregnancy [20, 21]. However, when APS presents with abdominal symptoms, the liver is most often affected by hepatic-veno-occlusive disease, while very little is known regarding adenomas [21]. Our patients reported no thromboembolic or bleeding events in her history, and had no prior pregnancies. After careful consideration, a high-prophylactic dosage was eventually chosen. Placement of a cava filter to prevent subsequent thromboembolisms from the subumbilical region was ultimately abandoned owing to the absence of deep venous thrombosis and our caution to not disturb the fragile equilibrium the patient had achieved.

## Conclusion

A hepatocellular adenoma is a rare diagnosis that requires centralized expertise when presenting with symptoms, such as abdominal bleeding. This particular case illustrates the complexity of diagnostic and treatment strategies for (active) bleeds from an adenoma, and concomitant conditions and complications. Although adenomas are often an incidental finding without induced symptoms, it can also result in significant morbidity when presenting with concomitant intra-abdominal bleeding. However, initial presentation of APS as multiple bleeding adenomas is very rare. Centralization of treatment leads to expertise in managing complex treatment strategies.

## Data Availability

All the patient data that was necessary to write this case report was available in the patient register of the Erasmus MC and was viewed and used with permission from the patient.
